# Fetal cord plasma herpesviruses and preeclampsia: an observational cohort study

**DOI:** 10.1038/s41598-024-65386-6

**Published:** 2024-06-25

**Authors:** Inka Häkkinen, Gamze Yazgeldi Gunaydin, Lari Pyöriä, Shohei Kojima, Nicholas Parrish, Maria F. Perdomo, Juho Wedenoja, Klaus Hedman, Seppo Heinonen, Eero Kajantie, Hannele Laivuori, Juha Kere, Shintaro Katayama, Satu Wedenoja

**Affiliations:** 1 Stem Cells and Metabolism Research Program, University of Helsinki, Helsinki, Finland; 2 Folkhälsan Research Center, Helsinki, Finland; 3 Department of Virology, University of Helsinki and Helsinki University Hospital, Helsinki, Finland; 4 Genome Immunobiology RIKEN Hakubi Research Team, RIKEN Center for Integrative Medical Sciences and RIKEN Cluster for Pioneering Research, Yokohama, Japan; 5 Department of Ophthalmology, University of Helsinki and Helsinki University Hospital, Helsinki, Finland; 6 Obstetrics and Gynecology, University of Helsinki and Helsinki University Hospital, Helsinki, Finland; 7 Public Health Promotion Unit, Finnish Institute for Health and Welfare, Helsinki and Oulu, Finland; 8 PEDEGO Research Unit, MRC Oulu, Oulu University Hospital and University of Oulu, Oulu, Finland; 9 Department of Obstetrics and Gynecology, Tampere University Hospital, Wellbeing Services County of Pirkanmaa, Tampere, Finland; 10 Faculty of Medicine and Health Technology, Center for Child, Adolescent and Maternal Health Research, Tampere University, Tampere, Finland; 11 Medical and Clinical Genetics, University of Helsinki and Helsinki University Hospital, Helsinki, Finland; 12 Department of Biosciences and Nutrition, Karolinska Institutet, Huddinge, Sweden; 13 Information Services Department, Finnish Institute for Health and Welfare, Helsinki, Finland

**Keywords:** Preeclampsia, Herpesvirus, DNA, Cord plasma, Biobank, Translational research, Herpes virus

## Abstract

A previous study suggested that fetal inheritance of chromosomally integrated human herpesvirus 6 (ici-HHV6) is associated with the hypertensive pregnancy disorder preeclampsia (PE). We aimed to study this question utilizing cord plasma samples (n = 1276) of the Finnish Genetics of Preeclampsia Consortium (FINNPEC) cohort: 539 from a pregnancy with PE and 737 without. We studied these samples and 30 placentas from PE pregnancies by a multiplex qPCR for the DNAs of all nine human herpesviruses. To assess the population prevalence of iciHHV-6, we studied whole-genome sequencing data from blood-derived DNA of 3421 biobank subjects. Any herpes viral DNA was detected in only two (0.37%) PE and one (0.14%) control sample (OR 2.74, 95% CI 0.25–30.4). One PE sample contained iciHHV-6B and another HHV-7 DNA. The control’s DNA was of iciHHV-6B; the fetus having growth restriction and preterm birth without PE diagnosis. Placentas showed no herpesviruses. In the biobank data, 3 of 3421 subjects (0.08%) had low level HHV-6B but no iciHHV-6. While iciHHV-6 proved extremely rare, both fetuses with iciHHV-6B were growth-restricted, preterm, and from a pregnancy with maternal hypertension. Our findings suggest that human herpesviruses are not a significant cause of PE, whereas iciHHV-6 may pose some fetal risk.

## Introduction

Preeclampsia (PE), a pregnancy complication affecting 2–8% of all pregnancies, remains a substantial public health problem and a major cause of maternal morbidity and mortality worldwide^1–3^. PE is characterized by maternal hypertension with abrupt onset after 20 weeks of gestation, accompanied by proteinuria or other related symptoms. Especially early onset PE with severe features often involves signs of placental insufficiency and fetal growth restriction^4^.

Beyond its root placental etiology, the pathogenesis of PE remains poorly understood^5^. The hallmarks of PE are immune activation, systemic inflammation, and endothelial dysfunction in the mother^6,7^. Notably, a substantial body of work points to association with infectious pathogens. Risk factors of placental changes resembling those of PE include influenza A exposure in rodents^8^, and in humans, many common bacteria, viruses, and parasites^9^. Among pathogenic viruses, those occurring most commonly at the maternal–fetal interface of the placenta are members of the *Herpesviridae* family. While their clinical and biological relevance remains poorly understood, especially human herpesvirus 6 (HHV-6A and -6B) might contribute to reproductive disorders including PE, unexplained primary infertility, and spontaneous abortion^10–17^.

Unlike other herpesviruses, HHV-6 can integrate into the human genome and pass from parent to child as inherited chromosomally integrated (iciHHV-6)^18,19^. A recent study suggested that placental RNA transcription of iciHHV-6 increases the risk of PE, and accordingly, fetal inheritance of iciHHV-6 shows a 2- to threefold risk of developing PE during pregnancy^20^.

We aimed to investigate the suggested link between PE and fetal exposure to herpesviruses. We searched for human herpesviruses from umbilical cord plasma of fetuses born from pregnancies with or without PE using a multiplex quantitative PCR. Moreover, we studied a set of placental samples for human herpesviruses and assessed the prevalence of iciHHV-6 in the Finnish population using whole-genome sequencing data from a national biobank.

## Methods

### Study population

The participants of this study belong to the Finnish Genetics of Preeclampsia Consortium (FINNPEC) cohort. This series recruited altogether 2206 pregnant women from all five Finnish university hospitals, located in Helsinki, Tampere, Turku, Kuopio, and Oulu. The recruitment process and sample retrieval have been described^20^. Once a woman with PE had been recruited, another woman attending the same hospital without PE was recruited as a control subject. The participants were initially recruited by research nurses and doctors responsible for the study in the participating hospitals. Exclusion criteria were multiple pregnancy and maternal age < 18 years. The study protocol was approved by the coordinating Ethics Committee of the Hospital District of Helsinki and Uusimaa (149/EO/2007) on October 7, 2007 (updated May 16, 2018). Research was performed in accordance with the approved guidelines and written informed consent was obtained from each participant.

As participants in the PE case–control cohort, both nulliparous and multiparous women with a singleton pregnancy were included. PE was defined according to the 2002 criteria of the American College of Obstetricians and Gynecologists (ACOG) as hypertension and proteinuria recorded after 20 gestation weeks^21^. Proteinuria was defined as urinary excretion of ≥ 0.3 g protein in a 24-h specimen, or 0.3 g/L, or two ≥ 1 + readings on a dipstick in at least one random urine sample, with no signs of urinary tract infection. For hypertension, a limit of ≥ 140 mm Hg for systolic and ≥ 90 mm Hg for diastolic pressure was used. PE was categorized as early onset if the diagnosis took place before gestation weeks 34 + 0 (weeks + days) and late onset if at gestation weeks 34 + 0 weeks or later. The delivery was defined as preterm when before 37 + 0 gestation weeks. The Finnish standards were used for small-for-gestational age (SGA): birth weight below − 2.0 SD^22^. Placental insufficiency was defined as the umbilical artery pulsatility index (PI) or resistance index (RI) >  + 2 SD^23^.

### Samples

Whole-blood cord samples were collected at delivery into EDTA tubes. Genomic DNA was extracted from 2 mL of umbilical cord plasma using the NucleoSpin Blood XL DNA extraction kit (Macherey–Nagel GmbH and Co) and elution volume of 200 μl. DNA was stored at − 20 °C. For qPCR analysis, DNA samples were diluted to 50 ng/μl.

Placental biopsies consisted of villous tissue under the basal plate. They had been obtained soon after delivery and stored snap-frozen in − 80 °C. DNA extraction was performed using the DNeasy Blood & Tissue Kit (Qiagen) according to the standard protocol. For qPCR, placental DNA samples were diluted to 50 ng/μl.

### Analysis by qPCR

The samples were analyzed using a DNA-qPCR (HERQ-9) targeting, differentiating and quantifying all nine human hepesviruses^24^. The multiplex approach comprises three triplex qPCRs: (i) herpes simplex viruses 1 (HSV1) and 2 (HSV2), and varicella zoster virus (VZV); (ii) Epstein-Barr virus (EBV), cytomegalovirus (CMV), and Kaposi’s sarcoma herpesvirus (KSHV); (iii) human herpesvirus 6A and 6B (HHV-6A and HHV-6B), and human herpesvirus 7 (HHV-7). In brief, the triplex runs were executed using AriaMx’s real-time PCR system and analyzed with their respective software. Water served as a negative control, and for each run, a standard curve was created with the corresponding triple-plasmid combination as a positive control diluted serially from 10^6 to 10^1. The numbers of viral copies in cord plasma were calculated as copies/mL.

### Biobank data analysis

National whole-genome sequencing (WGS) data from blood-derived DNA of the FINRISK Study^25^ included 3421 participants. WGS datasets achieved at least 19.5 × coverage per sample^26^. The data access and processing were approved by THL Biobank (study number THLBB2021_44). Biobank analyses were performed according to the approved guidelines and regulations as defined in the national Biobank Act (688/2012). Informed consent had been obtained from all subjects and/or their legal guardians.

The integrated_HHV6_recon tool (tag v231003 with the DIGEST 8c9968964d18; Docker system version: 20.10.24) was employed to extract unmapped reads from CRAM files and map them onto HHV-6A and HHV-6B genomes^27^. The human reference genome file (https://ftp.1000genomes.ebi.ac.uk/vol1/ftp/technical/reference/GRCh38_reference_genome/GRCh38_full_analysis_set_plus_decoy_hla.fa, last modified on 09.03.2015) was sourced from the International Genome Sample Resource^28^ on November 23, 2023. The virus reference genome file was readily available within the pipeline image. Default parameters were applied to the analysis. The analysis involved two outcomes: “no virus detected” and “virus detected”. In the absence of a virus, a 0.00% overall alignment rate indicated no alignment with the virus reference genome. For paired reads, when there were no discordant alignments, and all mate pairs exhibited zero alignment, data confirmed the absence of virus-related sequences. Conversely, in the presence of viral reads, the majority showed zero concordant alignments, a notable fraction aligned once, and a minor fraction aligned more than once. This resulted in a non-zero overall alignment rate, contrasting with 0.0%. These metrics provided the basis for interpreting virus detection in the WGS data.

### Statistical analysis

The results were analyzed by a two-tailed Fisher exact test and odds ratios provided with 95% confidence intervals for the association between PE and herpesviruses. The analyses were performed with Microsoft Excel (2021) version 16.56.

### Ethics approval

The study protocol was approved by the coordinating Ethics Committee of the Hospital District of Helsinki and Uusimaa (149/EO/2007) on October 7, 2007 (updated May 16, 2018). Study was performed in accordance with the approved guidelines and written informed consent was obtained from each participant. Biobank analyses were performed according to the approved regulations as defined in the national Biobank Act (688/2012). Informed consent had been obtained from all subjects and/or their legal guardians.

## Results

Among the 1276 pregnant women with cord plasma samples available, 539 had PE (median age of 29 y, range 18–47) and 737 were controls (median age 30 y, range 18–45). The demographics of the study population are shown in Table 1. The body mass index (BMI) and underlying diseases were similar in the two groups. The proportion of primiparas was somewhat higher in the PE than the control group, in line with the predilection of PE toward first pregnancies^1^.

**Table 1:** Demographics of the study population.

		Total	PE	Control
Participants	n (%)	1276 (100)	539 (42.2)	737 (57.8)
Age at birth of the child	Median (range)	29 (18–47)	29 (18–47)	30 (18–45)
BMI before pregnancy	Median (range)	23.3 (16.2–48.4)	23.9 (16.2–48.4)	23 (17.5–42.9)
Parity				
Primipara	n (%)	826 (64.7)	415 (77.0)	411 (55.8)
Multipara	n (%)	450 (35.3)	124 (23.0)	326 (44.2)
Family history of PE	n (%)	73 (5.7)	47 (8.7)	26 (3.5)
Underlying diseases				
Diabetes mellitus type 1	n (%)	6 (0.5)	1 (0.2)	5 (0.7)
Essential hypertension	n (%)	45 (3.5)	28 (5.2)	17 (2.3)
Chronic kidney disease	n (%)	5 (0.4)	2 (0.4)	3 (0.4)

PE = preeclampsia. BMI = body mass index.

Of the 539 PE cord plasma samples, only one (0.19%) harboured HHV-6B DNA and another, HHV-7 DNA (0.19%). Among the 737 controls, one (0.14%) had HHV-6B DNA. No PE or control sample contained DNA of any other human herpesvirus. The DNA findings among the cases and controls lacked statistical significance (Table 2).

**Table 2:** Findings of human herpesviruses by qPCR.

	Total n = 1276 n (%)	PE n = 539 n (%)	Control n = 737 n (%)	p-value	Odds ratio (95% CI)
**Any herpesvirus**	**3 (0.24)**	**2 (0.37)**	**1 (0.14)**	**0.62**	**2.74 (0.25–30.4)**
HSV-1	0 (0)	0 (0)	0 (0)	N/A	
HSV-2	0 (0)	0 (0)	0 (0)	N/A	
VZV	0 (0)	0 (0)	0 (0)	N/A	
EBV	0 (0)	0 (0)	0 (0)	N/A	
CMV	0 (0)	0 (0)	0 (0)	N/A	
HHV-6A	0 (0)	0 (0)	0 (0)	N/A	
**HHV-6B**	**2 (0.16)**	**1 (0.19)**	**1 (0.14)**	N/A	
**HHV-7**	**1 (0.08)**	**1 (0.19)**	0 (0)	N/A	
KSHV	0 (0)	0 (0)	0 (0)	N/A	

PE = preeclampsia. HSV = herpes simplex virus. VZV = varicella-zoster virus. EBV = Epstein-Barr virus.

CMV = cytomegalovirus. HHV = human herpes virus. KSHV = Kaposi’s sarcoma herpesvirus = HHV-8.

The clinical data of the three herpesvirus-positive cases are shown in Table 3. In the PE group, the participant with HHV-6B-containing fetal cord plasma was a 29-year-old primipara with normal BMI and no diabetes or kidney disease. There was no history of PE among relatives. PE was of early onset, at gestational week 28, and with severe features. She was recorded with placental insufficiency. Her infant, born prematurely at gestational age 29 weeks, showed growth restriction (− 2.8 SD). The cord plasma had 7.10 × 10^7^ copies/mL of HHV-6B.

**Table 3:** Clinical features of pregnancies with herpesviruses detected from fetal cord plasma.

		HHV-7 positive PE case	HHV-6B positive PE case	HHV-6B positive control
Age at birth	Years	22	29	22
BMI before pregnancy	kg/m^2^	21.7	19.8	28.5
Early onset PE	Yes/no	No	Yes	No
Severe PE	Yes/no	No	Yes	No
Primipara	Yes/no	Yes	Yes	Yes
PE in previous pregnancy	Yes/no	No	No	No
Family history of PE	Yes/no	No	No	No
Underlying diseases				
Diabetes mellitus type 1	Yes/no	No	No	No
Essential hypertension	Yes/no	No	No	No
Chronic kidney disease	Yes/no	No	No	No
Gestational				
Gestational hypertension	Yes/no	Yes (PE)	Yes (PE)	Yes
Gestational diabetes	Yes/no	No	No	No
Preterm delivery	Yes/no	No	Yes	Yes
Birth weight	SD	0.02	− 2.8	− 2.5
Placental insufficiency	Yes/no	No	Yes	Yes
Number of viral copies in cord plasma	Copies/mL	1.95 × 10^4^	7.10 × 10^7^	3.51 × 10^7^

PE = preeclampsia. BMI = body mass index. SGA = small for gestational age.

The PE case with HHV-7 was a 22-year-old primipara with normal BMI and no diabetes or kidney disease. There was no history of PE among relatives. Her PE showed no severe features and was late-onset. Her infant born on gestational week 38 was of average birthweight (0.02 SD). The cord plasma had 1.95 × 10^4^ copies/mL of HHV-7.

The control participant with HHV-6B was a 22-year-old primipara with a BMI of 28.5 kg/m^2^, and no diabetes or kidney disease. There was no history of PE among relatives. She had no pre-pregnancy hypertension and had hypertension during pregnancy but without a diagnosis of PE. She was recorded with placental insufficiency and her infant was SGA (− 2.5 SD) and was born prematurely at 31 weeks of gestation. The cord plasma had 3.51 × 10^7^ copies/mL of HHV-6B.

No parental samples were available for study of HHV-6B inheritance in these two fetuses. However, the likelihood of iciHHV-6 correlates strongly with viral load; HHV-6B DNA levels exceeding 500 000 copies/mL plasma are considered strongly suggestive of this condition^29^. The diagnosis can be confirmed with a ratio of viral to human genomes approaching 1:1^29^. The qPCR in our control showed a high HHV-6B level (3.51 × 10^7^ copies/mL), pointing to iciHHV-6B. In our PE patient, the HHV-6B level was even higher (7.10 × 10^7^ copies/mL DNA) and consistent with iciHHV-6B.

All studied 30 placental samples from PE pregnancies were negative for all nine human herpesviruses.

In the biobank WGS data, only 3 subjects (0.08%) of 3421 were positive for HHV-6. The corresponding HHV-6B genome alignment rates were 0.33%, 7.23%, and 27.3%, These low rates of mapped reads point to circulating HHV-6B and exogeneous infection, rather than iciHHV-6.

## Discussion

Many studies have suggested association between herpesviruses and pregnancy complications. In our series, only three cord plasma samples were found positive for any human herpesvirus DNA. Two of these fetuses, one with iciHHV-6B and another with HHV-7, were born from PE pregnancies. The third fetus, having iciHHV-6B, was found in a mother with hypertension and placental insufficiency but no formal diagnosis of PE at the time. However, as this control subject meets the latest criteria for PE^4^, where proteinuria is no longer required for diagnosis if signs of organ dysfunction and/or intrauterine growth restriction exist, her suitability for a healthy control is arguable. Even if this control would had been included among the cases with PE, no statistically significant difference for the HHV prevalence in PE and controls would have been detected. Collectively, the prevalence of herpesvirus DNA (0.2%) in our clinical cohort appeared somewhat lower than anticipated based on earlier literature: e.g. congenital HHV-6 DNA has been demonstrated in around 0.2–0.8% of newborns^30,31^. Strikingly, iciHHV-6B proved extremely rare among Finns and remained absent in 3421 Finnish biobank subjects. Both our data and the literature suggest that human herpesviruses—also HHV-6—in newborns are quite rare, and unlikely to represent a major cause of PE.

In our series, only one newborn among 539 (0.2%) PE pregnancies was positive for iciHHV-6B, and none of the PE placentas showed any human herpesvirus. In a study showing an association between HHV-6 and PE, iciHHV-6 was found in 2.1% (10 out of 467 cases) of fetuses from PE pregnancies and 0.8% (30 out of 3,854 cases) from control pregnancies^14^. While iciHHV-6 acts as a latent form of HHV-6, it can produce whole viruses^18,19^. Since herpesviruses persist in the body, their impacts on pregnancy outcomes cannot be excluded if encountered as latent. After primary HHV-6 infection, and febrile illness in children, the virus remains latent and persists in various tissues such as peripheral mononuclear cells, salivary glands, and the female genital tract^32–34^. Indeed, the prevalence of HHV-6 and HHV-7 latent infections is high among pregnant women. Caserta et al. found 26% of PBMC samples HHV-6-DNA-positive during the first half of pregnancy and 22% during the second half. HHV-7 was even more common, with 64% of samples positive during the first half and 67% during the second. In the cord plasma samples analyzed in the same study, two out of five HHV-6 positive samples were positive for iciHHV-6. None of the cord plasma samples were positive for HHV-7^11^. These data indicate that despite the high prevalence of latent infections, the rate of transmission to the fetus is low, consistent with our cord plasma results.

A major challenge in showing association between viral infections and pregnancy complications is the timing of sample retrieval and the possibility of maternal, placental, and fetal infections. Placental or cord plasma samples negative for viral DNA after delivery cannot rule out latent infection or infection during early pregnancy. Another challenge is that PE is a heterogeneous disorder, overlapping in presentation with other pregnancy complications^1^. Indeed, the control newborn positive for iciHHV-6B in our series was born from a non-PE pregnancy that showed, however, gestational hypertension, placental insufficiency, SGA infant with preterm birth at 31 weeks. According to the current criteria, however, these features are consistent with the diagnosis of PE^4^. Thereby, the data suggest that iciHHV-6B might have contributed to pregnancy complications in that case, even if the diagnostic criteria of PE were not met at the study entry. HHV-6 infects many cells including endometrial, epithelial, and endometrial natural killer (eNK) cells, as well as syncytiotrophoblasts^16,35,36^. A recent study suggested that an HHV-6A infection of endometrial cells could cause inadequate trophoblast adhesion to endometrial cells through modified expression of miRNAs^37^. Therefore, it is not surprising that HHV-6 is the herpesvirus repeatedly linked to reproductive failure and different pregnancy complications^15^.

None of our samples were positive for any other herpesvirus DNAs apart from HHV-6B and HHV-7. Yet again, while facing the issue of rarity of transplacental transmission, it could be argued that since our sample size was considerable, an association between PE and an in utero herpesvirus infection is highly improbable. In agreement with this conclusion, previous studies have failed to consistently link herpesviruses other than HHV-6, such as HSV-2^38^ or CMV^39^, to PE.

The main limitation of our study was still the relatively limited number of participants, and the lower than anticipated number of viral hits. The estimated rate for HHV-6 DNA in cord plasma at birth is around 1%. Of these, 86% are caused by the infant’siciHHV-6, 10% by transplacental transmission of viruses produced by the mother’s iciHHV-6, and 4% by a maternal acute HHV-6 infection via transplacental transmission^40^. These findings and the estimated rates of HHV-6 DNA in newborns of around 1%^30,31^, in fact, mean that a larger series might give a statistically significant difference between PE and controls. Even in that case, however, the prevalence of a transplacental iciHHV-6B acquisition would remain low and its clinical impact limited.

Unfortunately, we had no placental or parental samples available from the HHV-6B positive fetuses. However, the high copy numbers of HHV-6B in cord plasma are suggestive of fetal iciHHV-6B and possibly also of reactivation. Our findings support previous research by Gaccioli et al., who showed a correlation between fetal iciHHV-6 and placental transcription of viral RNA to preeclampsia^14^.

One question relates to the methodological aspects of our study. We chose to use qPCR to identify possible herpesvirus infections for two reasons. The method is sensitive and fast. In addition, the use of cord plasma further reduces the possibility of coming across a latent infection. Due to the low number of cord plasma samples positive for herpesviruses in the present study, we screened a small set of placental DNA samples from PE pregnancies, without viral hits however. Moreover, we strengthened the analysis using the whole genome sequencing data of 3421 individuals from national biobank, demonstrating no viral hits and suggesting a very low prevalence of iciHHV-6B among Finns. This contrasts with the frequency of 0.8% in the UK blood donor data^41^ and suggests that iciHHV-6B is not evenly distributed across European-ancestry populations.

Overall, there still is no evidence for an association between PE and herpesviruses apart from HHV-6. In our data with over 500 PE pregnancies, cord plasma herpesvirus DNA findings proved rare. The only herpesviruses we found were HHV-6B and HHV-7, with prevalences too low for evidence of PE association. An interesting finding was the major difference in iciHHV-6 prevalence between Finnish and British populations. Collectively, our data do not definitively overrule the possibility that in those few fetuses with iciHHV-6 there might be a link to PE. Major population differences in iciHHV-6 carriership but similar incidence of PE suggest, however, that this factor is not epidemiologically substantial.

## Data Availability

The datasets analysed during the current study are not publicly available because the patients were not consented for publishing the data, but are available from the corresponding author on reasonable request.
